# One- and 2-year flare rates after treat-to-target and tight-control therapy of gout: results from the NOR-Gout study

**DOI:** 10.1186/s13075-022-02772-3

**Published:** 2022-04-20

**Authors:** Till Uhlig, Lars F. Karoliussen, Joe Sexton, Tore K. Kvien, Espen A. Haavardsholm, Fernando Perez-Ruiz, Hilde Berner Hammer

**Affiliations:** 1 Division of Rheumatology and Research, Diakonhjemmet Hospital, Box 23, Vinderen, N-0319 Oslo, Norway; 2 Faculty of Medicine, University of Oslo, Oslo, Norway; 3 Osakidetza, OSI EE-Cruces, Division of Rheumatology, Cruces University Hospital, Baracaldo, Spain; 4 Biocruces-Bizkaia Health Research Institute, Baracaldo, Spain; 5 Medicine Department, Medicine School, University of the Basque Country, Leioa, Spain

**Keywords:** Gout, Treat to target, Flare, Urate lowering treatment, Predictor

## Abstract

**Objectives:**

To explore the frequency and predictors of flares over 2 years during a treat-to-target strategy with urate-lowering therapy (ULT) in patients with gout.

**Methods:**

In the treat-to-target, tight control NOR-Gout study patients started ULT with escalating doses of allopurinol. Flares were recorded over 2 years. Baseline predictors of flares during months 9–12 in year 1 and during year 2 were analyzed by multivariable logistic regression.

**Results:**

Of 211 patients included (mean age 56.4 years, disease duration 7.8 years, 95% males), 81% (150/186) of patients experienced at least one gout flare during the first year and 26% (45/173) during the second year. The highest frequency of flares in the first year was seen during months 3–6 (46.8% of patients).

Baseline crystal depositions detected by ultrasound and by dual-energy computed tomography (DECT) were the only variables which predicted flares both during the first period of interest at months 9–12 (OR 1.033; 95% CI 1.010–1.057, and OR 1.056; 95% CI 1.007–1.108) and also in year 2. Baseline subcutaneous tophi (OR 2.42, 95% CI 1.50–5.59) and prior use of colchicine at baseline (OR 2.48, 95% CI 1.28-4.79) were independent predictors of flares during months 9–12, whereas self-efficacy for pain was a protective predictor (OR 0.98 per unit, 95% CI 0.964–0.996).

**Conclusions:**

In patients with gout, flares remain frequent during the first year of a treat-to-target ULT strategy, especially during months 3–6, but are much less frequent during year 2. Baseline crystal depositions predict flares over 2 years, supporting ULT early during disease course.

**Trial registration:**

ACTRN12618001372279

## Background

Gout is the most prevalent inflammatory arthritis [1]. The disease is characterized by acute episodes of debilitating pain and joint inflammation, which current nomenclature defines as gout flares [2], with a wide variation in the pattern of flare over time [3]. Gout confers an increased mortality as compared to the general population [4].

Recurrent gout flares are associated with reduced health-related quality of life (HRQoL) and work participation [5, 6], and gout flares are also endorsed by OMERACT as a core outcome domain in long term clinical trials [7]. A patient-reported definition of flare has been suggested [8] and validated [9].

Higher serum urate (SUA) levels and longer disease duration of gout have been considered to carry an elevated risk for acute gout flares, but there is variability with other factors involved, leaving us with limited knowledge on prognostic factors for recurrent gout flares [10].

Long-term use of urate-lowering therapy (ULT) leads to crystal dissolution, reduces the risk of flare [11], and prevents joint damage [12]. Recommendations suggest considering initiation with ULT already close to the time of diagnosis to reduce the frequency of gout flares and morbidity [13, 14]. Gout flares are common after initiation of ULT [15], and therefore prophylactic treatment with colchicine or non-steroidal anti-inflammatory drugs (NSAID for 3–6 months after start with ULT is recommended [13, 14] to reduce new flares [16–18].

Given sparse evidence regarding factors associated with gout flare during initiation and escalation of ULT in patients, we studied the incidence of gout flares over 2 years follow-up during ULT and examined predictors of flares in gout.

## Methods

### Study design and participants

NOR-Gout (Gout in Norway) is a prospective, observational single-center study in a hospital-based rheumatology unit. Patients were eligible if having a gout attack within the last month, had increased SUA (> 360 μmol/L), and no contraindication for ULT. Other severe co-morbidities including chronic kidney disease stage 3b and higher were exclusion criteria. Patients were consecutively included according to the protocol (ACTRN12618001372279). In all patients, a diagnosis of gout was based on identification of monosodium urate crystals in polarized microscopy after arthrocentesis [19] performed by a rheumatologist. The study had been approved by the regional ethics committee, included patient representatives in project planning, and was performed in accordance with the declaration of Helsinki. All patients provided written informed consent. The sponsor of the study was Diakonhjemmet Hospital.

### Treatment

Patients received at baseline individual information by trained research nurses on gout, including non-pharmacological and pharmacological management. Drug use was recorded for NSAID, colchicine, prednisolone, and for ULT (allopurinol, febuxostat) at every visit which also included registration of drug dosage and adverse events. All patients not already on ULT started as recommended [13, 20] with oral allopurinol 100 mg once per day and escalated by 100 mg increments monthly according to SUA concentrations until a maximum of 900 mg daily. If there is intolerance for allopurinol, febuxostat was started at 40 mg once daily and escalated monthly to 80 and 120 mg as needed. Probenecid or lesinurad could be added if necessary but were not used in any patients. Patients received flare prophylaxis, with prescribed colchicine 0.5–1 mg daily, individualized for 3–6 months, as recommended for the first months in current EULAR recommendations in 2015 when the study was initiated [20]. In this treat-to-target approach, ULT was escalated to reach a serum urate target level of < 360 μmol/L (or < 300 μmol/L if clinical tophi were present), and the dose was maintained when the target was reached.

### Visits

A study nurse and a rheumatologist (HBH, LK) (who also performed ultrasound) assessed patients at baseline as well as after 3, 6, 12, and 24 months. Additional scheduled visits with only the study nurse were at 1, 2, and 9 months, and if necessary monthly, until the treatment target was reached. Telephone contact with review of the SUA result could substitute for face-to-face visits. During the second year, patients were followed by their general practitioners as needed.

### Flare definition

A gout flare during months 9–12 in year 1 was the primary clinical outcome. At every clinical visit during the 2-year study, the patient self-reported gout flares since the last visit during a structured interview with the study nurse who recorded the flares. If in doubt, the patient and study nurse discussed whether an experienced episode with pain or swelling was to be defined as a gout flare or not.

At baseline, self-reported information on number of flares ever and during the last year before study entry was collected by questionnaire as well as pain severity during the most recent and the strongest attack (0–10 numerical rating scales), with 0 = no pain and 10 = unbearable pain. Flares were reported as the frequency of patients having had ≥ 1 flare as well as the total number of flares, at all study time points, as recommended [21]. The number of self-reported flares with joint swelling in the previous year and also in total before the study was categorized into 0, 1, and 2–5.

### Covariates

#### Demographics and self-reported measures

At baseline, patients reported age, gender, ethnicity, marital status, family history of for gout, disease duration, highest level of education, comorbidities, and working status. For comorbidities, the Self-Administered Comorbidity Questionnaire (SCQ) was used (range 0–36) [22]; it includes 12 medical problems, allocating 1 point per problem including presence, receiving treatment, and causing a functional limitation.

Daily and previous smoking, consumption of alcohol and sugar sweetened drinks, and the frequency of physical activity were reported by patients.

#### Questionnaires

Questionnaires at each visit recorded present joint pain due to gout, general pain, fatigue, and patient global assessment of disease activity, all on 0–10 numerical rating scales.

Physical function was measured with the Health Assessment Questionnaire (HAQ) without adjustment for help or devices [23]. Health status was assessed by the Short Form general health questionnaire (SF-36) [24].

Self-efficacy with subscales for pain (5 items) and symptoms (6 items) was measured with the Arthritis Self-Efficacy Scales [25]. This instrument measures whether patients have confidence in coping with pain, function, and other symptoms due to arthritis (numeric rating scales 10–100, 100 = highest).

The Beliefs about Medicines Questionnaire (BMQ) [26] explores patients’ beliefs about medicines and includes scales on perceived necessity or concerns for the patient’s own medicines (5 items each, range 5–25) and for perceived general overuse and harm of medicines (4 items each with range 4–16). Items were scored on Likert scale 1–5, 5 = highest agreement, and a high scale score reflects stronger belief in the expressed concept.

#### Clinical assessments

Clinical assessments included weight and height for calculation of body mass index (BMI) and 44-swollen and tender joint counts and clinical examination for subcutaneous tophi.

#### Imaging with ultrasound and dual-energy computed tomography (DECT)

To assess the level of crystal deposition, all patients were examined by ultrasound and DECT. Ultrasound was at baseline scored as previously described [27] (score 0-3 of double contour, tophi and aggregates) with calculation of total ultrasound sum scores.

DECT baseline scoring of feet and ankles applied the semiquantitative Bayat method (scores 0–3) [28, 29], and a sum score for the four regions (first metatarsophalangeal joint, other joints of the toes, ankles and midfeet, and tendons) was derived.

#### Laboratory assessments

SUA was analyzed at each study visit and is presented both as a dichotomous (cutoff 360 μmol/L) and continuous variable, as recommended [21]. Laboratory examinations included SUA (μmol/L), erythrocyte sedimentation rate (ESR) mm/h, C-reactive protein (CRP) mg/L, creatinine (μmol/L), and eGFR (ml/min/1.73m^2^, CKD-EPI formula) at baseline and follow-up visits.

### Statistics

Descriptive measures of baseline variables are presented using frequency, mean, and standard deviation. Differences between groups with and without flares during defined time periods were explored using independent sample *T*-test and by the *χ*^2^ test or Fisher’s exact, as appropriate. A cumulative probability plot shows flares in the first year, where every patient is one observation, and flares are ordered from 0 to maximum flare number.

Odds ratio (OR) with 95% confidence intervals (95% CI) were calculated by logistic regression analyses after performing bivariate analyses with baseline candidate predictor variables of flares at 9–12 months and during the second year. These variables were selected from baseline data, based on their potential clinical relevance, and were in case of possible statistical relevance (*p* < 0.10) then entered in stepwise backwards multivariable logistic regression analyses, adjusting for age and gender and disease duration and retained if statistically significant (*p* < 0.05). Analyses were performed with IBM SPSS statistics (version 27).

## Results

### Patient characteristics

Of 211 patients, 186 completed follow-up at year 1 (88.2%) [19] and 173 patients (82.0%) at year 2. No statistical differences were observed between baseline characteristics in 2-year completers versus non-completers.

SUA decreased from mean 500 μmol/L at baseline to 311 μmol at 1 year and 324 μmol/L at year 2, and 85.5% of patients were at target < 360 μmol at year 1 and 78.6% at year 2. Demographics and baseline characteristics are shown in Table 1 for all patients, and for those with and without flares during months 9–12 in years 1 and during year 2. Patients were predominantly middle-aged men with a mean disease duration of around 8 years, and 16.6% had subcutaneous tophi.

**Table 1:** Baseline characteristics for all patients, and patients with and without a flare (percentage or mean with standard deviation (SD)

	Baseline		1 year follow-up (*N* = 186)			2 year follow-up (*N* = 173)		
			No flare Months 9–12	Flare Months 9–12	*p*-value	No flare Year 2	Flare Year 2	*p*-value
	***N*** = 211		***N*** = 116	***N*** = 70		***N*** = 128	***N*** = 45	
Age (years)	211	56.4 (13.7)	56.3 (13.8)	57.6 (13.2)	0.52	56.8 (13.9)	57.1 (12.9)	0.88
Male	201/211	95.3%	96.6%	92.9%	0.30	93.8%	97.8%	0.45
Caucasian	183/202	90.6%	89.5%	92.4%	0.51	92.0%	86.0%	0.25
Disease duration (years)	204	7.8 (7.6)	7.4 (6.8)	9.6 (9.3)	0.10	8.1 (8.2)	8.8 (6.3)	0.62
College education	118/206	57.3%	61.7%	58.2%	0.64	58.3%	66.7%	0.34
Married/cohabiting	155/208	74.5%	76.7%	77.6%	0.89	76.4%	76.7%	0.96
Working	133/208	63.9%	69.8%	61.2%	0.23	64.1%	73.8%	0.25
Body mass index (kg/m^2^)	211	28.8 (4.5)	28.5 (4.6)	29.7 (4.6)	0.09	29.2 (4.5)	28.1 (4.7)	0.18
Co-morbidities (SCQ sum)	210	3.7 (3.2)	3.3 (1.1)	4.5 (3.4)	**0.013**	3.7 (3.3)	3.8 (3.4)	0.91
Physical activity ≥ 3 times weekly	163/207	30.4%	36.8%	26.5%	0.15	29.1%	41.9%	0.12
Smoking, daily	23/208	11.1%	10.3%	4.5%	0.16	10.9%	2.4%	0.12
Alcohol consumption at least weekly	128/207	61.8%	61.2%	62.1%	0.90	63.0	57.1%	0.61
Sugar sweetened drinks daily	80/207	38.6%	40.5%	33.3%	0.34	38.6%	38.1%	0.96
≥ 1 subcutaneous tophus present	35/211	16.6%	12.1%	24.3%	**0.017**	14.1%	22.2%	0.20
Allopurinol use ever	31/211	14.7%	13.8%	14.3%	0.92	14.8%	6.7%	0.16
NSAID use ever	160/205	78.0%	74.3%	88.1%	**0.028**	77.6%	86.0%	0.23
Colchicine use ever	107/201	53.2%	43.8%	67.7%	**0.002**	50.4%	56.1%	0.53
Prednisolone use ever	91/199	45.7%	47.3%	53.1%	0.46	45.1%	52.4%	0.41
Baseline SUA (μmol/L)	211	500 (77)	491 (81)	510 (78)	0.12	496 (81)	504 (81)	0.58
ESR (mm/h)	199	14 (14)	14 (13)	15 (15)	0.46	14 (14)	17 (15)	0.21
Creatinine (μmol/L)	211	96 (18)	96 (17)	96 (19)	0.90	95 (17)	99 (20)	0.11
eGFR (ml/min. per 1.73 m^2^)	210	78 (19)	78 (18)	77 (18)	0.71	78 (18)	75 (18)	0.27
Previous flares					**0.042**			0.10
0	16	7.7%	6.0%	9.0%		7.8%	7.1%	
1	25	12.0%	16.4%	3.0%		12.5%	2.4%	
2-5	65	31.3%	29.3%	28.4%		32.0%	23.8%	
> 5	102	49.0%	48.3%	49.7%		47.7%	66.7%	
Previous flares during last 12 months	151/206	73.4%	73.7%	77.6%	0.38	69.3%	90.5%	0.053
Strongest joint pain ever (0–10)	208	8.4 (1.6)	8.1 (1.6)	8.7 (1.4)	**0.013**	8.2 (1.6)	8.7 (1.1)	0.051
Joint pain last flare (0–10)	207	7.5 (5.5)	7.1 (2.0)	7.1 (2.1)	0.96	7.1 (2.1)	7.3 (1.9)	0.51
Swollen joint present	72/209	34.4%	33.8%	37.7%	0.60	31.0%	44.4%	0.10
Tender joint present	110/210	52.4%	47.8%	62.3%	0.06	50.0%	57.0%	0.37
Health assessment questionnaire (0–3)	209	0.38 (0.57)	0.33 (0.58)	0.41 (0.44)	0.35	0.34 (0.54)	0.43 (0.69)	0.35
SF-36 physical component summary (0–100)	204	39 (11)	40 (11)	36 (10)	**0.006**	40 (10)	37 (12)	0.21
SF-36 mental component summary (0–100)	204	50 (10)	50 (10)	50 (10)	0.91	51 (10)	49 (10)	0.31
Self-efficacy pain (10–100)	209	65 (19)	68 (19)	61 (20)	**0.015**	65 (19)	64 (21)	0.69
Self-efficacy symptoms (10–100)	205	72 (15)	73 (17)	72 (18)	0.51	73 (17)	72 (15)	0.84
Beliefs about Medicines Questionnaire								
Necessity subscale (5–25)	198	17.9 (4.4)	16.8 (4.2)	17.2 (4.5)	0.54	16.8 (4.3)	17.0 (4.6)	0.99
Concerns subscale (5–25)	197	13.4 (4.9)	12.8 (4.5)	14.2 (4.1)	0.053	13.3 (4.5)	14.0 (4.1)	0.30
Overuse subscale (4–16)	203	10.6 (2.8)	10.5 (2.9)	10.7 (2.5)	0.57	10.6 (2.8)	10.8 (2.7)	0.57
Harm subscale (4–16)	203	9.4 (2.4)	9.3 (2.5)	9.5 (2.3)	0.68	9.4 (2.5)	9.5 (2.1)	0.66
Ultrasound sum score	209	20.0 (13.9)	17.4 (12.0)	23.7 (15.6)	**0.004**	18.8 (13.0)	23.5 (14.0)	**0.036**
Dual energy computed tomography sum score	187	4.6 (6.4)	3.8 (6.0)	6.2 (7.0)	**0.019**	4.0 (5.9)	6.7 (7.5)	**0.037**

*SCQ* Self-Administered Comorbidity Questionnaire, *NSAID* non-steroidal anti-inflammatory drug, *ESR* erythrocyte sedimentation rate, *eGFR* electronic glomerular filtration rate, *SF-36* Short-form 36

All patients initiated or escalated ULT. Only 14.7% (31/211) of patients had ever used ULT with allopurinol and none had used febuxostat, while 78% had experience ever with NSAID, and about half with each colchicine and prednisolone. During the first year, prescription of allopurinol decreased from 95.0% to 87.6% due to switch to febuxostat and increased for febuxostat from 3.5 to 12.4%. Mean doses for allopurinol remained just below 300 mg and below 60 mg for febuxostat. Flare prophylaxis with colchicine was used by 76.3% (161/211) of patients from baseline, with 72.3% (146/202) using colchicine at 1 month, 75.6% (146/193) at 2 months, 42.8% (80/189) at 3 months, and 14.5% (27/187) at 6 months follow-up. NSAIDs and prednisolone were not used as prophylaxis for flares.

### Flares

**Fig. 1:**
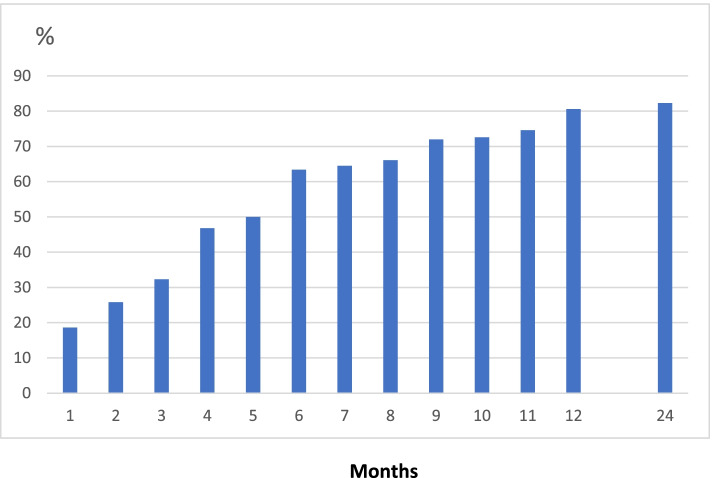
Cumulative incidence of flares during year 1 and after 2 years (*n* = 186)

In the first year, 80.6% (150/186) of patients experienced at least one gout flare and 26.0% (45/173) during the second year. The cumulative incidence of flares during the study is shown in Fig. 1. The mean number of flares was 2.7 (SD 2.8) during the first year and 0.7 (SD 2.19) during year 2 (median 2 and 0, respectively). Flares before study entry had been experienced by 92.3% of patients and more than five flares by 49% (Table 1), and 73.4% of patients had experienced at least one other flare in the last year before inclusion.

**Fig. 2:**
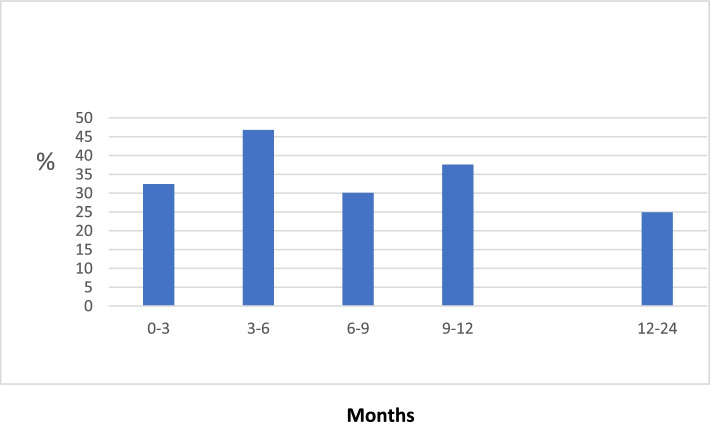
Flare frequency during the 3 months periods in year 1 and in year 2 after treat-to-target ULT

Table 2 gives incidence numbers of flare per month, aggregated for 3-month periods and cumulatively during the first year in patients with at least one flare. The flare frequency in year 1 was highest during months 3–6 (46.8%) and was in the following 3-month periods between 30.1% and 37.6% (Fig. 2).

**Table 2:** Flares in 1-year completers (*n* = 186) per month and in intervals

	Flare last month or since last examination		Cumulative flares	
	*N*	%	*N*	%
Month 1	34/183	18.6	34/186	18.3
Month 2	26/176	14.8	48/186	25.8
Month 3	32/178	18.0	60/186	32.3
Month 4	39/164	23.8	87/186	46.8
Month 5	17/70	24.3	93/186	50.0
Month 6	60/182	33.0	118/186	63.4
Month 7	14/55	25.5	120/186	64.5
Month 8	10/42	23.8	123/186	66.1
Month 9	34/166	20.5	134/186	72.0
Month 10	16/59	27.1	135/186	72.6
Month 11	4/27	14.8	135/186	72.6
Month 12	57/186	30.6	150/186	80.6
Months 0–3	59/186	31.7		
Months 3–6	87/186	46.8		
Months 6–9	56/186	30.1		
Months 9–12	70/186	37.6		
Months 0–12	150/186	80.6

Characteristics for SUA, drug use, and flare history are displayed in Table 3 for patients with flares in the 3-month periods of year 1 and during year 2. Patients with and without flares were over time not consistently statistically different for demographic and disease-related factors.

**Table 3:** Characteristics of patients with at least one flare during 3-month periods and during year 2

	Year 1				Year 2
	0–3 months	3–6 months	6–9 months	9–12 months	
*N* with flare	59/189	90/187	52/167	70/186	45/173
Age (years)	56.0 (13.9)	56.9 (13.0)	59.5 (14.7)	57.6 (13.2)	57.1 (12.9)
Disease duration (years)	8.4 (8.6)	8.4 (7.3)	10.2 (9.8) *	9.6 (9.3)	8.8 (6.3)
Baseline > 1 tophus	16.9%	14.4%	19.2%	24.3% *	22.2%
Co-morbidities (SCQ) sum	4.6 (3.9) *	4.0 (3.4)	4.5 (3.4)	4.5 (3.4)*	3.8 (3.4)
Baseline SUA	502 (76)	504 (76)	498 (81)	510 (38)	504 (81)
3 months SUA	361 (76) *	343 (62)	339 (61)	345 (60)	351 (68)
6 months SUA	325 (65)	328 (61)	335 (63)*	331 (63)	331 (66)
9 months SUA	312 (54)	317 (61)	318 (69)	328 (60)	311 (58)
12 months SUA	304 (47)	309 (46)	299 (47)	308 (48)	313 (51)
24 months SUA	330 (80)	331 (79)	323 (68)	337 (85)	328 (67)
Baseline allopurinol user (%)	15.3%	17.8%	13.5%	14.3%	6.7%
Month 3 allopurinol (mg)	238 (86)	239 (98)	224 (105)	253 (97)*	228 (105)
Month 6 allopurinol (mg)	287 (98)	275 (120)	272 (134)	308 (124)**	275 (124)
Month 9 allopurinol (mg)	292 (128)	281 (133)	293 (145)	307 (137)*	291 (146)
Month 12 allopurinol (mg)	303 (126)	295 (135)	301 (146)	333 (142)**	292 (138)
Ever use					
- NSAID	87.5%	79.3%	80.4%	88.1%*	86.0%
- Colchicine	52.7%	60.0%	57.7%	67.7%*	56.1%
- Prednisolone	51.8%	53.6%	56.9%	53.1%	52.4%
≥ 1 flare last 12 months	82.5%	82.4%	88.5%	77.6%	90.5%
> 5 previous flares	59.6%	56.8%	63.5%*	59.7%	52.4%*
Strongest pain ever	8.7 (1.3)*	8.5 (1.5)	8.6 (1.2)	8.7 (1.4)	8.7 (1.1)
Strongest pain last flare	7.1 (2.0)	7.2 (2.0)	7.4 (2.0)	7.1 (2.1)	7.3 (1.9)

*p-value < 0.01 and with higher value compared to non-flare group

**p-value < 0.01 and with higher value compared to non-flare group

*SUA* serum urate, *NSAID* non-steroidal anti-inflammatory drug

**Fig. 3:**
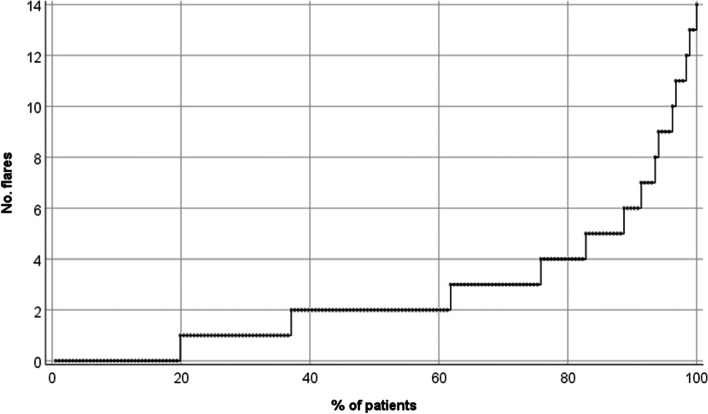
Cumulative probability plot for number of flares during the first 12 months of treat-to-target ULT (*n* = 186). Every patient is represented by one dot, sorted from low to high

The distribution of flares during year 1 among patients is presented as a cumulative probability plot in Fig. 3, demonstrating the median number of flares to be two, and 10% of patients had six or more flares.

### Prediction of flares

Measures for baseline urate deposition (clinical tophi, ultrasound and DECT measures) were all bivariately related to flares in year 1 (months 9–12), but baseline ultrasound and DECT sum scores were the only variables which were associated with flares in year 2. There was no consistent relationship between other variables and flares at year 2, including SUA levels or allopurinol dose. For months 9–12, some other baseline factors were significantly associated with flares in bivariate analyses: more co-morbidities, more frequently experience with NSAID and colchicine ever, more flares before study entry, higher pain during the worst flare ever, worse physical function (SF-36 physical component summary), and lower self-efficacy (Table 1).

In multivariable logistic regression analyses with adjustment for age, gender, and disease duration, only baseline ultrasound and DECT sum scores were consistent predictors of flares, both during months 9–12 and year 2 (Table 4). Tophaceous disease was an independent predictor for flares during months 9–12, in addition to self-efficacy of pain and previous experience with colchicine, but none of these predicted flares during year 2.

**Table 4:** Prediction of flares during time periods by baseline variables using logistic regression

	Flare year 1 (months 9–12)		Flare year 2	
	Unadjusted OR (95% CI), *p*-value	Adjusted OR (95% CI) ^a^, *p*-value	Unadjusted OR (95% CI), *p*-value	Adjusted OR (95% CI) ^a^, *p*-value
Ultrasound sum score	1.034 (1.011–1.058) *p* = 0.004	1.033 (1.010–1.057) *p* = 0.005	1.025 (1.001–1.050) *p* = 0.040	1.027 (1.00–1.55) *p* = 0.049
DECT sum score	1.059 (1.010–1.111) *p* = 0.017	1.056 (1.007–1.108) *p* = 0.026	1.062 (1.009–1.117) *p* = 0.021	1.064 (1.003–1.029) *p* = 0.035
Subcutaneous tophus	2.34 (1.07–5.10) *p* = 0.033	2.42 (1.50–5.59) *p* = 0.038	1.75 (0.74–4.13) *p* = 0.20	–
Self-efficacy for pain (10–100)	0.981 (0.965–0.997) *p* = 0.017	0.980 (0.964–0.996) *p* = 0.017	0.998 (0.981–1.016) *p* = 0.83	–
Colchicine ever used at baseline	2.69 (1.42–5.11) *p* = 0.002	2.48 (1.28–4.79) *p* = 0.007	1.26 (0.62–2.56) *p* = 0.53	–

^a^Adjusted for age, gender, and disease duration

*DECT* dual-energy computed tomography

Neither baseline SUA nor final ULT dose with allopurinol after 1 and 2 years were associated with incidence of a new flare during months 9–12 or year 2. Further, no other demographic or life-style characteristics predicted gout flares.

In sensitivity analyses, we examined the relationship between previous ULT and flares and stratified also for patients who still used prophylaxis after 3 and 6 months. No relationship for previous ULT and flares was observed. There was a higher frequency of flares during months 9–12 in patients using prophylaxis at months 3 versus not (49.4% vs. 25.3%, *p* < 0.001), but not for flares in year 2. Prophylaxis status at month 6 was not related to flares during months 9–12 or year 2.

## Discussion

This study examined over 2 years flare frequency and predictors of flares in gout patients actively treated with ULT. Four out of five patients experience a flare during year 1 but only one of four during year 2. Flares were seen most frequently in patients during months 3–6 (46.8%).

Importantly, crystal depositions at baseline were evaluated by three methods (subcutaneous tophi, ultrasound and DECT), and all three methods could predict flares at months 9–12 and ultrasound and DECT also at year 2. This is a novel finding, and determination of the crystal load by three methods and over 2 years in this study strengthens the validity of findings. Crystal depositions are only slowly resolved during therapy, and therefore, flares must be expected in patients with a high crystal burden.

We also found that patients with high self-efficacy for gout pain independently had a lower risk for flares during months 9–12, whereas patients with previous experience with colchicine at baseline had an increased risk of flares. We have earlier shown in NOR-Gout that high self-efficacy contributes to achieving the target SUA level at 1 year [19].

Patients with frequent flares may have used colchicine more frequently both before and during the first months of the study. It could thus be that colchicine use in this study is more an indicator of frequent flares and disease severity, and our non-randomized design does not allow to study the prevention of flares with colchicine.

Interestingly, no other demographic, life-style factors, SUA, or medication predicted flares in our study. While high SUA does increase gout incidence and flare recurrence [30], no relevant relationship between low SUA and flares was found in a systematic review [31] based on RCTs, whereas results from the extension studies indicated that lowering and maintaining serum urate to < 360 μmol/L was associated with some reduced occurrence of gout flares, in line with some other studies [12, 16, 32]. Thus, the association between low SUA levels and reduction in flares seems weak. Flares have also been associated with decreases and fluctuations in urate levels in response to pegloticase treatment [33], a finding which supports the hypothesis that not momentary SUA levels, but rather fluctuations, could initiate an inflammatory process manifested as a flare.

Other studies find frequent flares early after initiating ULT [3, 34] or over time [32] and especially during the first 3–6 months after initiating ULT [15, 35]. In a recent randomized controlled trial, gout flares were increased in the active ULT arm even increased during the first year but reduced in year 2 as compared to the usual care arm [36]. We report a high frequency of flares during all quarters of the first year, but mainly during months 3–6 where many patients no longer used prophylactic treatment with colchicine. We set flares during months 9–12 as the primary clinical outcome, expecting that after ambitious ULT the SUA levels had by then been low and stable for some time. In our study, we planned for patients to receive prophylactic colchicine only for the first few months as previously recommended [20], but treatment was not strictly supervised and only a minority of patients were still using colchicine at 6 months as recommended in the most recent EULAR recommendations from 2016 [13]. The observed high frequency of flares during months 3–6 supports consistent flare prophylaxis after ULT.

Absence of consistent clinical predictors of flares was also observed in a long-term evaluation after the incidence of gout [37]. Other studies find that alcohol consumption [38] and co-morbidities such as hypertension and diabetes are associated with more flares [39]. In patients with a gout flare during a hospital stay, flares can be predicted based on factors observed before admission [40].

The reporting of flares in clinical studies of gout has not been standardized and various methods have been used. Flare in gout shows a high variation [3], and there are challenges with flare reporting, including the quality of flares [21]. Lack of a standardized and validated flare definition prevents comparisons and within-group discrimination [41] but can now be overcome with a validated method for self-report [9].

Our study is large and with frequent follow-up visits, showing that while the promoted urate target is realistic in daily clinical practice, gout flares must be expected.

Limitations in our study include the single-center design. Secondly, flare assessment was mainly self-reported, and the study was initiated before publication of validated self-reported flare criteria [9]. Thirdly, recall bias most likely affected reported flares, especially during year 2, which included no study visits between 12 and 24 months follow-up. A patient diary for flare reporting could have overcome recall bias. However, the consultation with study nurses at the 2-year visit gave an opportunity to recall flares the last year. Finally, the observational nature and lack of a control group in our study does not allow causal inferences.

Our study finds frequent flares with increasing cumulative incidence during the first year, even though ULT lead to low SUA levels already after 3–4 months [19]. Four out of five patients must expect at least one flare during the first year of ULT, but flares are clearly less frequent during the second year. The degree of crystal depositions at baseline was found to be associated with the frequency of flares during the two years, supporting that ULT needs to be optimized to achieve the treatment target and remove depositions. Further research should apply a validated definition of flares and investigate if flares decrease in strength and duration during treat-to-target ULT.

## Conclusions

In conclusion, patients with gout frequently flare during the whole first year, especially during months 3–6, but flares are much less frequent during year 2 when treated with ULT. Baseline crystal depositions predict flares over 2 years, supporting ULT early during disease course.

## Data Availability

The datasets used during the current study are available from the corresponding author on reasonable request.

## Abbreviations

BMI: Body mass index
BMQ: Beliefs about Medicines Questionnaire
CI: Confidence interval
CRP: C-reactive protein
DECT: Dual-energy computed tomography
ESR: Erythrocyte sedimentation rate
HAQ: Health Assessment Questionnaire
HRQoL: Health-related quality of life
NOR-Gout: Gout in Norway
NSAID: Non-steroidal anti-inflammatory drugs
OMERACT: Outcome measures in rheumatoid arthritis clinical trials
OR: Odds ratio
SCQ: Self-Administered Comorbidity Questionnaire
SD: Standard deviation
ULT: Urate-lowering therapy
